# Dysregulation in cortical reactivity to emotional faces in PTSD patients with high dissociation symptoms

**DOI:** 10.3402/ejpt.v4i0.20430

**Published:** 2013-09-04

**Authors:** Aleksandra Klimova, Richard A. Bryant, Leanne M. Williams, Kim Louise Felmingham

**Affiliations:** 1 School of Psychology, University of New South Wales, Australia; 2 Brain Dynamics Centre, Westmead Millenium Institute, Western Clinical School, University of Sydney, Australia; 3 School of Psychology, University of Tasmania, Australia

**Keywords:** posttraumatic stress disorder (PTSD), dissociation, ERPs, faces, emotion

## Abstract

**Background:**

Predominant dissociation in posttraumatic stress disorder (PTSD) is characterized by restricted affective responses to positive stimuli. To date, no studies have examined neural responses to a range of emotional expressions in PTSD with high dissociative symptoms.

**Objective:**

This study tested the hypothesis that PTSD patients with high dissociative symptoms will display increased event-related potential (ERP) amplitudes in early components (N1, P1) to threatening faces (angry, fearful), and reduced later ERP amplitudes (Vertex Positive Potential (VPP), P3) to happy faces compared to PTSD patients with low dissociative symptoms.

**Methods:**

Thirty-nine civilians with PTSD were classified as high dissociative (*n*=16) or low dissociative (*n*=23) according to their responses on the Clinician Administered Dissociative States Scale. ERPs were recorded, whilst participants viewed emotional (happy, angry, fear) and neutral facial expressions in a passive viewing task.

**Results:**

High dissociative PTSD patients displayed significantly increased N120 amplitude to the majority of facial expressions (neutral, happy, and angry) compared to low dissociative PTSD patients under conscious and preconscious conditions. The high dissociative PTSD group had significantly reduced VPP amplitude to happy faces in the conscious condition.

**Conclusion:**

High dissociative PTSD patients displayed increased early (preconscious) cortical responses to emotional stimuli, and specific reductions to happy facial expressions in later (conscious), face-specific components compared to low dissociative PTSD patients. Dissociation in PTSD may act to increase initial pre-attentive processing of affective stimuli, and specifically reduce cortical reactivity to happy faces when consciously processing these stimuli.

Despite the growing recognition of the role of dissociation in posttraumatic stress disorder (PTSD), dissociative symptoms have received relatively little empirical attention. Dissociation refers to the disruption and fragmentation of the usually integrated function of consciousness, memory, identity, body awareness, and perception of self and the environment (American Psychiatric Association, 2000). Dissociative PTSD symptoms, such as depersonalization and derealization (Murray, Ehlers, & Mayou, 2002), contribute significantly to functional impairment in PTSD (Norman, Stein, & Davidson, 2007). To date, there are very few investigations of neural processes associated with dissociative symptoms in PTSD. This study examined neural processes associated with high and low dissociative symptoms in PTSD using event-related potentials (ERPs), which provide a high temporal resolution measure of cortical processing.

Whilst the function of dissociation is still debated, several theories propose that dissociation may be a regulatory strategy invoked in response to severe emotional arousal or threat (Holmes et al., 2005). Consistent with this proposal, functional magnetic resonance imaging (fMRI) studies reveal that PTSD patients with predominant dissociative responses to a trauma narrative display *increased* activation in prefrontal regulatory networks (specifically medial prefrontal gyrus and anterior cingulate cortex) compared to the typically reduced activity in these prefrontal networks seen in PTSD patients with predominant hyperarousal responses (Felmingham et al., 2008; Lanius et al., 2002).

The proposal that dissociation is an avoidant strategy invoked by extreme arousal would suggest that high dissociative symptoms in PTSD would be associated with greater early reactivity to threat stimuli. Facial stimuli displaying different emotional expressions were used as a salient stimulus to examine dissociation. Initial fMRI evidence in line with this proposal has been reported, as amygdala and brainstem arousal networks were found significantly activated in response to subliminally presented fearful faces in PTSD patients with who reported high dissociative responses compared to those with low dissociative responses to subliminally presented fearful faces (Felmingham et al., 2008). As this greater limbic and brainstem activity was observed in response to *subliminally* presented fearful faces, it was interpreted to reflect greater automatic (preconscious) arousal activation.

However, fMRI studies are limited by poor temporal resolution; therefore, the timing of the effect of dissociation on neural processes can only be inferred. It is possible that dissociation involves a shift between reactivity states (from automatic preconscious arousal to conscious regulation), which cannot be adequately investigated using methodologies with poor temporal resolution such as fMRI. ERPs provide a high temporal resolution index of phasic cortical reactivity that more precisely reflects pre-conscious and conscious processing. Given their high temporal resolution, ERPs are a useful technique to discriminate early preconscious emotional reactivity from conscious emotion regulation.

An early ERP components (N120: negative-going waveform approximately 100 ms post-stimulus) is thought to reflect sensory processing and has been found to be modulated by arousal and preconscious attention processes (Hillyard, Teder-Salejarvi, & Munte, 1998; Naatanen, 1990). In terms of ERPs to emotional faces, there is evidence that early components (around 100–120 ms post-stimulus) reflect rapid emotional processing of faces (Eimer & Holmes, 2002; Pizzigalli, Regard, & Lehmann, 1999) and are associated with increased engagement of autonomic arousal processes toward emotional stimuli (Lithari et al., 2010; Olofsson, Nordin, Sequeira, & Polich, 2008). Numerous ERP studies to facial stimuli have reported evidence of conscious face-specific processing components in temporal/occipital regions (the N170) and at medial sites (the Vertex Positive Potential (VPP), a positive-going waveform around 200 ms post-stimulus; Eimer & Holmes, 2002; Felmingham, Bryant, & Gordon, 2003). A recent study of the temporal nature of ERPs found evidence for phased processing of facial emotions, including an early detection system at around 100 ms post-stimulus which directed processing resources to salient stimuli, and then more detailed conscious processing at around 170 ms post-stimulus which enable discrimination of facial expressions (Utama, Takemoto, Koike, & Nakamura, 2009). Finally, the P300 component (positive-going waveform approximately 300 ms post-stimulus) is thought to be a measure of cognitive processing as it varies in amplitude with attention, memory, and stimulus categorization (Polich & Kok, 1995). P3 amplitude is generally elicited in response to emotional stimuli that involve some motivational significance and/or task relevance (Polich & Kok, 1995).

Therefore, ERPs to facial emotions provide an excellent methodology to examine the timing of the influence of dissociative symptoms on neural processes. Specifically, if dissociation results in early preconscious reactivity to emotional stimuli, it would be predicted that the high dissociation PTSD group would display greater amplitudes in early ERP components (N1) compared to the low dissociation group. Second, if dissociation then results in conscious regulation of emotional processing, it would be predicted that a high dissociation PTSD group would display reduced amplitudes in later ERP components to facial emotions.

To date, no studies have examined ERPs in response to facial emotional expressions in regard to dissociation and PTSD. Most ERP studies in PTSD have used attention tasks, and have reported reduction in P3 amplitudes which have been interpreted to reflect deficits in attention allocation in PTSD (Felmingham, Bryant, Kendall, & Gordon, 2002; McFarlane, Weber, & Clark, 1993). Only two previous ERP studies have examined dissociation in PTSD, and both have used attentional tasks. Both studies reported that high dissociation symptoms in PTSD were associated with reduced P3 amplitudes in an attention task, suggesting that increased dissociation is associated with impaired attention (Felmingham et al., 2002; Kimble, Fleming, & Bandy, 2010). Given that a key aspect of dissociation in PTSD is reduced reactivity to positive signals and reduced experience of positive emotions (such as love and happiness), examining ERP responses to emotional stimuli (and in particular, positive emotion stimuli) is particularly important. Previous emotional ERP studies in PTSD have reported dysregulation in ERP components (N1, N2, and P3) in response to angry, sad, or happy faces (Ehlers et al., 2006; Felmingham et al., 2003), but they did not examine the effect of dissociation symptoms.

The majority of studies examining dissociation and PTSD have used threatening stimuli (fearful faces or trauma narratives), and there is very little evidence examining the impact of dissociative symptoms in PTSD on the range of emotional responses. Furthermore, to examine the effects of dissociation on preconscious and conscious processes, it would be useful to examine ERPs in response to both subliminally presented faces (reflecting preconscious processing) and supraliminal faces (reflecting conscious processing).

Accordingly, this study will examine the effect of dissociative symptoms in PTSD on ERP responses to a range of emotional facial expressions, including anger, fear, happy, and neutral expressions, under supraliminal (conscious) and subliminal (preconscious) conditions. This study will investigate the effect of dissociative symptoms on early ERP components (N1) to provide an index of early preconscious emotional reactivity, and on later ERP components (the VPP, P3) to provide an index of conscious emotional processing of faces. We will test the predictions that PTSD participants with high dissociative symptoms will reflect increased early ERP responses (increased N1 amplitude), and reduced later ERP responses (VPP and P3 amplitude) to emotional faces compared to PTSD participants with low dissociative symptoms. Second, it is predicted that the increased N1 amplitude in the high dissociation group will be particularly evident to threatening facial expressions (anger, fear) under subliminal (preconscious) conditions. Finally, it is predicted that the reduced later ERP components (VPP, P3) in the high dissociation group will be particularly evident in response to positive facial stimuli (happy faces) in the conscious processing condition.

## Method

### Participants

The sample consisted of 39 participants diagnosed with PTSD. Participants were diagnosed by clinical psychologists using the Clinician Administered PTSD Scale (Blake et al. 1990). PTSD participants had experienced a range of traumatic events, including motor vehicle accidents (MVAs) and falls (*n*=16), physical assault (*n*=16), natural disasters (*n*=1), police duties (*n*=5), and sexual assault (*n*=1). Traumatic events had occurred at least 3 months prior to the study, and the average time post-trauma was 4.3 years (SD=5.8, range: 5 months to 20 years). The average total CAPS score was 72.5 (SD=18), reflecting a severe level of PTSD. In order to be included in the study, participants had to: (a) meet the criteria for PTSD; (b) be aged between 18 and 65 years; and (c) have no history of a neurological disorder, brain injury, or substance abuse or dependence. Participants were classified as having high or low dissociative symptoms using the Clinician Administered Dissociative States Scale (CADSS: Bremner et al., 1998) which was administered after participants completed the facial expression viewing task. Following previous studies (Felmingham et al., 2008; Lanius et al., 2002), participants who scored below 15 were classified as low dissociative (*n*=23) and those scoring above 15 as highly dissociative (*n*=16). Written informed consent was obtained prior to participation. This study was approved by the Sydney West Area Health Services (SWAHS) Human Research Ethics committee.

### Materials and apparatus

Face stimuli were selected from a standardized set of facial emotion stimuli (Gur et al., 2002) that displayed neutral, happy, fearful, and angry expressions. Each face was a greyscale image, equated in terms of size and luminance.

### Study design

The study employed a 2 (group: PTSD_high dissociation/PTSD_low dissociation)×2 (condition: preconscious/conscious)×4 (valence: neutral, angry, happy, fear)×2 (site) mixed factorial design.

### Procedure

During ERP recording, blocks of eight stimuli per emotion were presented under both conscious (to elicit conscious, controlled processing) and preconscious (preconscious, to engage automatic processing) conditions. Each block contained eight stimuli, depicting fear, neutral, happy or angry emotions, presented in a pseudorandom order. There were four repeat blocks for each expression, making a total of 32 stimuli per expression. In the conscious condition, stimuli were presented for 500 ms. The inter-stimulus interval was 700 ms, making a total stimulus onset asynchrony of 1,200 ms.

In the preconscious condition, a backward masking protocol was used for stimuli presentation. Target stimuli (fear, angry, happy, and neutral) were presented for 10 ms, followed immediately by a neutral face mask presented for 150 ms. These durations were based on parameters established in a psychophysics experiment (Williams et al., 2004), and have been shown in previous analyses to prevent conscious detection of the stimulus. The masking face stimulus was superimposed over the target stimulus, but spatially offset by 1° in one of the four diagonals, randomly allocated. To ensure that preconscious and conscious conditions were equivalent, the ISI in the preconscious condition was 1,040 ms, making the total stimulus asynchrony 1,200 ms. Participants were instructed to pay attention to the faces in preparation for post-testing briefings, hence ensuring that participants attended to the stimuli.

### ERP data acquisition

Participants were required to sit in a sound and light attenuated room with temperature controlled at 24°C. They were asked to abstain from smoking or caffeinated drinks 2 hours prior to testing since these factors are known to affect ERPs. Data were acquired continuously at 500 Hz with skin resistance of <5 kΩ from 32 EEG channels, with four EOG channels to allow for detection of any eye movement artifacts, using a Quikcap and NuAmps system according to the International 10-10 electrode system. The sites of particular interest were those implicated in face processing (Eimer 2000; Felmingham et al., 2003) in temporal (left T5; right T6), frontal (Fz and Cz) and occipital (left O1; right O2) cortical areas. Data were recorded in relation to the virtual ground, and referenced offline to linked mastoids. Eye movement correction was undertaken offline using procedures from Gratton, Coles, and Donchin (1983).

### ERP data analysis

Average ERPs were calculated for each emotion stimuli. Individual single-trial ERP epochs were filtered with a low-pass Tukey (cosine) filter that attenuated frequencies above 25 Hz. Single trials were then averaged and peak components were identified within defined latency windows and validated by visual inspection across individual participants for each site. Data were collected from occipital and temporal regions, but elevated levels of artifact from poor electrode impedences at these sites precluded meaningful analysis. At the midline sites (Fz, Cz, Pz), the following components were examined: the N120 (maximum negative peak, 80–150 ms), VPP (VPP: maximum positive peak 120–220 ms), N200 (maximum negative peak 180–280 ms), and P300 (maximum positive peak 230–450 ms). ERP components were scored using a baseline to peak method. Outliers were defined as values exceeding 2.5 standard deviations from the mean, and formed only 1.5% of the data. These values were removed and replaced with group means.

Repeated-measures analyses of variance (ANOVAs) were used to analyze the amplitude of each component, with group (high dissociative PTSD vs. low dissociative PTSD) as the between-group factor, condition (conscious, preconscious), and emotion (fear, angry, happy, neutral) as within-subject factors. Analyses were conducted in separate 2 (Group)×2 (condition: conscious/preconscious)×4 (emotion: fear, angry, happy, neutral)×2 (sites: Fz, Cz) ANOVAs for each component. Clinical and demographic data were analyzed using one-way ANOVAs or Chi-squared tests where appropriate. A Greenhouse Geissner correction to degrees of freedom was used where there was sphericity in the data. An alpha value of *p*<0.05 was employed for all analyses, and *post-hoc* analyses were conducted using SIDAK tests.

## Results

### Demographic and clinical data

Table 1 displays the mean demographic and clinical data for each group. There were no significant differences in age, depression level, anxiety level, or stress level (using DASS scores), trauma type or time-post trauma, or total CAPS scores between the high and low dissociation groups. Chi-squared tests revealed there were significantly more women in the high compared to the low dissociation group (*χ* ^2^=5.6, *p*<0.05).

**Table 1:** Clinical and demographic data for the high and low dissociative PTSD groups

	Low dissociative	High dissociative	Test statistics	*p*
Age	42.5 (11.3)	41.4 (12.8)	0.08	0.8
Gender	10F/13M	13F/3M	5.6	0.018*
Depression	11.7 (6.2)	9.5 (6.4)	0.85	0.36
Anxiety	7.8 (5)	7.4 (5.3)	0.04	0.85
Stress	12.9 (4.4)	9.5 (5.4)	3.7	0.07
CAPS total	75.7 (17.8)	70.8 (18.7)	0.67	0.42
CADDS	6.8 (4.9)	24.5 (10.9)	46.9	0.000**
Education	13.6 (3.1)	12.9 (4.8)	0.28	0.6
Trauma type	13 MVA/10 Ass	7 MVA/9 Ass	0.62	0.52
Time—post (months)	58.5 (69)	42.5 (62)	0.55	0.46

NB: depression, anxiety and stress scores are subscale scores from the Depression Anxiety and Stress Scales. CAPS and CADDS are total scores on the CAPS and CADDS. F=female, M=male; MVA=motor vehicle accident, Ass=assault; PTSD=posttraumatic stress disorder.

Standard deviations are given in parentheses.

* *p*<0.05

** *p*<0.01.

### ERP data—Sites: Fz, Cz, and Pz

#### N120 amplitude

For N120, only the main effects of condition (*F*=6.57, *p=*0.015) and site (*F*=13.591, *p*=0.000) were found to be significant. A number of significant two-way interaction effects were observed. Mainly, interactions between emotion and group (*F*=2.92, *p*=0.042), condition and emotion (*F*=3.83, *p*=0.042) and condition and site were significant (*F*=7.677, *p*=0.002).

**Fig. 1:**
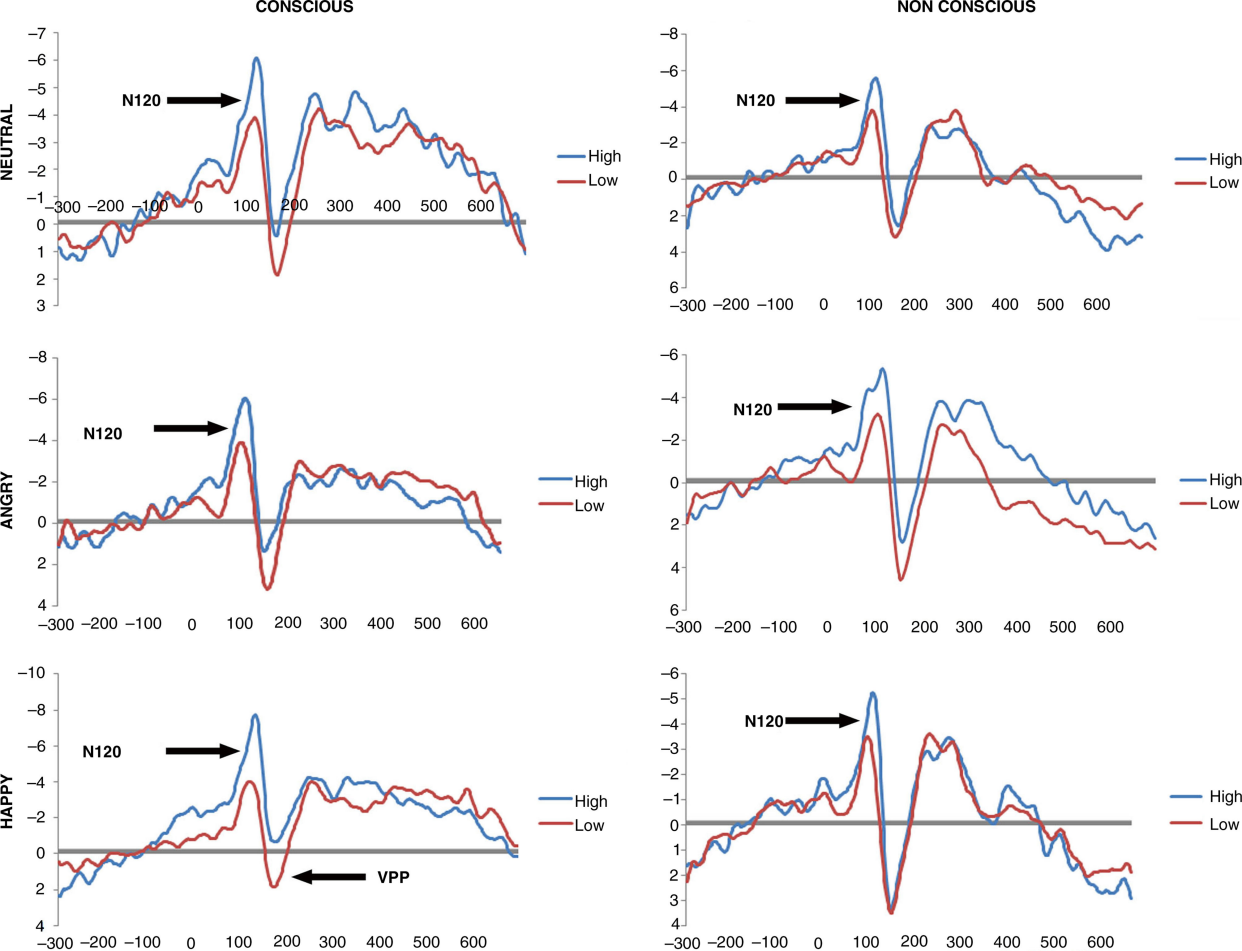
ERP in response to consciously and preconsciously processed neutral, angry, and happy faces.

A near-significant condition×emotion×group interaction effect was observed (*F*_(2.748,__38)_=2.75, *p*=0.051). Follow-up SIDAK *post-hoc* tests showed increased N120 amplitude in response to neutral (HD-LD=− 1.384, *p*=0.043 and HD-LD=− 2.431, *p*=0.008, respectively), happy (HD-LD=− 3.280, *p=*0.000 and HD-LD=− 1.763, *p*=0.012, respectively), and angry (HD-LD=− 2.678, *p*=0.006 and HD-LD=− 1.738, *p*=0.018, respectively) faces for the high compared to the low dissociative PTSD groups (see Fig. 1), indicating that the high dissociative group displayed significantly greater N120 amplitude compared to the low dissociative group at these valences. This effect was significant under both conscious and preconscious conditions, but was stronger under conscious conditions for happy and angry faces.

No other main effects or interactions were significant (see Supplementary Table 1).

#### VPP amplitude

For VPP amplitude, the main effects of condition (*F*=32.598, *p*=0.000) and emotion (*F*=8.792, *p*=0.000) reached significance. The only significant two-way interaction was observed between condition and site (*F*=10.714, *p*=0.001).

A significant condition×emotion×group interaction was obtained (*F*_(2.813,__38)_=2.925, *p*=0.041) for VPP amplitude. SIDAK *post-hoc* tests indicated that the high dissociation group showed reduced activation to consciously processed happy faces compared to the low dissociation group (Sidak *p*=0.027) (Fig. 1), but there were no significant group differences to happy faces in the preconscious condition (*p*>0.05) or to other facial expressions. No other main effects or interactions were significant (see Supplementary Table 2).

#### N200 amplitude

For N200, significant main effects were observed for site (*F*=34.4, *p*=0.000) and emotion (*F*=6.568, *p=*0.001, respectively) revealing a central maximum for N200 amplitude, and greater N200 amplitude to emotional than neutral faces. No other main effects or interaction effects were significant (see Supplementary Table 3 for details).

#### P300 amplitude

The main effects of condition (*F*=11.45, *p=*0.002), site (*F*=37.9, *p*=0.000), and emotion (*F*=8.38, *p*=0.000) were found to be significant, reflecting greater P3 amplitude in conscious compared to preconscious conditions, a parietal maximum, and greater P3 amplitude to the emotional compared to the neutral face. There were no other significant main or interaction effects (see Supplementary Table 4 for details).

#### Correlational analysis

To further investigate the relationship between dissociation scores and ERP components, which displayed significant differences between the dissociation groups, correlational analysis was conducted. For the N120 component, there was a significant negative correlation between N120 amplitude to happy faces and CADDS scores (conscious: *r*= − 0.64, *p*=0.000; preconscious: *r*= − 0.471, *p*=0.008), N120 amplitude to angry faces and CADDS scores (conscious: *r*= − 0.318, *p*=0.048; preconscious: *r*= − 0.34, *p*=0.034), and N120 amplitude to neutral faces and CADDS scores (conscious: *r*= − 0.547, *p*=0.000; preconscious: *r*= − 0.386, *p*=0.015). These correlations reveal that as CADDS scores increase, N120 amplitudes increase in response to angry, neutral, and happy faces under conscious and preconscious conditions. Furthermore, a significant negative correlation was observed between VPP amplitude to happy faces and CADDS scores (conscious: *r*= − 0.64, *p*=0.000; preconscious: *r*= − 0.326, *p*=0.043) reflecting reduced VPP amplitude to happy faces with increased CADDS scores.

## Discussion

This is the first study to our knowledge to examine ERPs to facial expressions in relation to dissociative symptoms in PTSD. As current models propose that dissociation is an avoidant regulatory strategy invoked in response to emotional arousal (Holmes et al., 2005), it was predicted that patients with high dissociative symptoms would greater early ERP (N1) amplitudes to threatening faces, and reduced later ERP (VPP, P3) responses to happy facial expressions compared to those with low dissociative responses. Our initial hypothesis was partially confirmed as the high dissociative PTSD group revealed increased N120 amplitudes to most facial stimuli, but we did not find that this was specific to threat stimuli. Confirming our third hypothesis, results revealed that PTSD patients with high dissociative symptoms displayed reduced later cortical activity (VPP) to happy facial expressions compared to low dissociative PTSD patients in conscious (but not preconscious) conditions.

The proposal that dissociation is an avoidant strategy invoked by extreme arousal would suggest that high dissociative symptoms would be associated with greater early reactivity to facial stimuli. In particular, it was predicted that the high dissociative PTSD group would display larger N1 and P1 amplitudes to threat stimuli (angry and fear faces) under preconscious conditions. This prediction was partially supported, as high dissociation PTSD patients displayed greater N120 amplitudes to angry, happy, and neutral faces compared to low dissociation PTSD patients, but this was particularly evident in the conscious condition processing angry and happy faces. It may be that happy and angry faces are the most salient emotional stimuli to a high dissociation PTSD group. It was somewhat surprising that we did not find enhanced N120 amplitudes to fearful facial expressions, as previous fMRI studies have revealed significant differences in high and low dissociative PTSD groups in response to fearful faces (Felmingham et al., 2008). One possible explanation for this is that fearful expressions are more complex and less directly threatening than angry faces, and require empathy on the part of the perceiver (Stemmler, Ave, & Wacker, 2007). This may lead to significantly greater variability amongst individuals in response to fearful faces, which may reduce the power to find effects. In addition, the early time frame of the N120 component may have led to reduced sensitivity to the fearful face than the other facial expressions.

N120 amplitude is thought to reflect sensory processing and have been found to be modulated by arousal and pre-conscious attention processes (Hillyard et al., 1998; Naatanen, 1990). In terms of ERPs to faces, there is evidence that early ERP components (around 110 ms post-stimulus) reflect rapid emotional processing of faces (Eimer & Holmes, 2002; Pizzigalli et al., 1999) and detection of facial expressions (Utama et al., 2009). Therefore, the increased N120 amplitudes to the majority of facial expressions in the high dissociation PTSD participants may reflect generalized increased arousal and pre-attentive processing. This is consistent with evidence that increased arousal results in greater automatic attention to salient emotional stimuli (Aston-Jones, Rajkowski, Kubiak, Valentino, & Shipley, 1996). It also accords with previous ERP findings in an American Indian sample with PTSD, which revealed increased N120 to sad facial expressions combined with an increase in preparatory gamma-band EEG signals (Ehlers et al., 2006). The N120 effect in this study was observed under both conscious and non-conscious conditions, but given the early nature of this component, it is considered to reflect an automatic attentional process.

The second key finding of this study was that the frontocentral VPP component (a concomitant of the face-specific N170 waveform over temporal–occipital sites) was significantly reduced specifically to happy faces in the high dissociation group under conscious conditions. This finding also confirmed our hypothesis that the high dissociative group would display reduced conscious processing of positive stimuli, and accords with recent findings of disturbed (slower) P3 processing of happy faces in an American Indian PTSD sample (Ehlers et al., 2006), and of reduced later processing of emotional faces in PTSD (Felmingham et al., 2003). However, these earlier ERP studies did not examine the impact of dissociation in PTSD. The prevailing theoretical model of emotional numbing suggests that hypervigilance for threat in PTSD leads to a predominance of processing resources devoted to threat stimuli, resulting in a reduction of conscious processing resources available for positive stimuli (Litz & Gray, 2002). The current finding of reduced VPP amplitude specifically for processing happy faces accords with this model. However, we did not find a concurrent increase in VPP amplitude to fearful or angry facial expressions.

A recent study of the temporal nature of ERPs found evidence for phased processing of facial emotions, including an early detection system at around 100 ms post-stimulus which directed processing resources to salient stimuli, and then more detailed conscious processing at around 170 ms post-stimulus which enable discrimination of facial expressions (Utama, et al., 2009). In light of this temporal model, our current findings may reflect an enhanced emotional detection system for facial expressions in the high dissociation group, but a specific reduction in processing happy faces with later more detailed conscious processing. The fact that we found a specific reduction in VPP amplitude to happy facial expressions reinforces the proposal that dissociation is associated with reduced reactivity to positive signals rather than a reduced reactivity to affect signals in general.

It is important to note that there were a significantly greater proportion of women in the high dissociative PTSD group compared to the low dissociative group, which may have confounded the results of our study. Although ERP studies have found that women have increased N2 and P3 amplitudes (Bianchin & Angrilli, 2011) and slow evoked potentials (Allison et al., 1994) to emotionally negative stimuli compared to men (Bianchin & Angrilli, 2011), there is no evidence to our knowledge revealing reduced amplitudes to happy facial expressions in women, or alterations in very early ERP components in women to emotional facial expressions. To rule out the impact of gender on our findings, future research needs to examine the impact of dissociative symptoms within larger samples of men and women with PTSD.

It was unfortunate that we could not assess cortical responses over temporal and occipital regions in the current study. Future research should increase the number of presentations of each facial stimulus, to ensure excellent signal-to-noise ratios and minimize the variability in the signal. Examining autonomic responses concurrently with ERPs would enable further examination of the relationship between arousal and early ERP processes.

In summary, this study provides evidence of increased pre-attentive cortical processing of facial emotional expressions and reduced later conscious processing of happy facial expressions in PTSD patients with high dissociative symptoms relative to those with low dissociative symptoms. This suggests that dissociative responses in PTSD may act to increase initial automatic processing of affective stimuli, and specifically reduce cortical reactivity to happy faces when consciously processing these stimuli. This evidence highlights the need for further ERP studies to identify the temporal sequencing of selective processing of emotional stimuli as a potential key mechanism in this subtype of PTSD.

## References

[CIT0001] Allison T, Ginter H, McCarthy G, Nobre A. C, Puce A, Luby M (1994). Face recognition in human extrastriate cortex. Journal of Neurophysiology.

[CIT0002] American Psychiatric Association (2000). Diagnostic and statistical manual of mental disorders.

[CIT0003] Aston-Jones G, Rajkowski J, Kubiak P, Valentino R, Shipley M (1996). Role of the locus coerulus in emotional activation. Progress in Brain Research.

[CIT0004] Bianchin M, Angrilli A (2011). Gender differences in emotional responses: A psychophysiological study. Physiology and Behaviour.

[CIT0005] Blake D. D, Weathers F. W, Nagy L. M, Kaloupek D. G, Klauminzer G, Charney D. S (1990). A clinician rating scale for assessing current and lifetime PTSD: The CAPS-1. Behaviour Therapy.

[CIT0006] Bremner J. D, Krystal J. H, Putnam F, Southwick S. M, Marmar C, Charney D. S (1998). Measurement of dissociative states with the Clinician Administered Dissociative States Scale (CADSS). Journal of Traumatic Stress.

[CIT0007] Ehlers C. L, Hurst S, Phillips E, Gilder D. A, Dixon M, Gross A (2006). Electrophysiological responses to affective stimuli in American Indians experiencing trauma with and without PTSD. Annals of New York Academy of Sciences.

[CIT0008] Eimer M (2000). The face-specific N170 component reflects late stages in the structural encoding of faces. Neuroreport.

[CIT0009] Eimer M, Holmes A (2002). An ERP study on the time course of emotional face processing. Neuroreport.

[CIT0010] Felmingham K. L, Bryant R. A, Gordon E (2003). Processing angry and neutral faces in post-traumatic stress disorder: An event-related potential study. Neuroreport.

[CIT0011] Felmingham K. L, Bryant R. A, Kendall C, Gordon E (2002). Event-related potential dysfunction in posttraumatic stress disorder: The role of numbing. Psychiatry Research.

[CIT0012] Felmingham K. L, Kemp A. H, Williams L. M, Falconer E. M, Olivieri G, Peduto A (2008). Dissociative responses to conscious and non-conscious fear impact underlying brain function in posttraumatic stress disorder. Psychological Medicine.

[CIT0013] Gratton G, Coles M. G, Donchin E (1983). A new method for off-line removal of ocular artefact. Electroencephalography and Clinical Neurophysiology.

[CIT0014] Gur R. C, Sara R, Hagendoorn M, Maron O, Hughgett P, Turner T. H (2002). A method for obtaining 3-dimensional facial expressions and its standardization for use in neurocognitive studies. Journal of Neuroscience Methods.

[CIT0015] Hillyard S. A, Teder-Salejarvi W. A, Munte T. F (1998). Temporal dynamics of early perceptual processing. Current Opinion in Neurobiology.

[CIT0016] Holmes E. A, Brown R. J, Mansell W, Fearon R. P, Hunter E. C, Frasquilho F (2005). Are there two qualitatively distinct forms of dissociation? A review and some clinical implications. Clinical Psychology Review.

[CIT0017] Kimble M, Fleming K, Bandy C (2010). Attention to novel and target stimuli in trauma survivors. Psychiatry Research.

[CIT0018] Lanius R. A, Williamson P. C, Boksman K, Densmore M, Gupta M, Neufeld R. W (2002). Brain activation during script driven imagery induced dissociation responding in posttraumatic stress disorder: A functional magnetic resonance imaging investigation. Biological Psychiatry.

[CIT0019] Litz B, Gray M. J (2002). Emotional numbing in posttraumatic stress disorder: Current and future directions. Australian and New Zealand Journal of Psychiatry.

[CIT0020] Lithari C, Frantzidis C. A, Papadelis C, Vivas A. B, Klados M. A, Kourtidou-Papdelis C (2010). Are females more responsive to emotional stimuli?: A neurophysiological study across arousal and valence dimensions. Brain Topography.

[CIT0021] McFarlane A. C, Weber D. L, Clark C. R (1993). Abnormal stimulus processing in posttraumatic stress disorder. Biological Psychiatry.

[CIT0022] Murray J, Ehlers A, Mayou R. A (2002). Dissociation and post-traumatic stress disorder: Two prospective studies of road traffic accident survivors. British Journal of Psychiatry.

[CIT0023] Naatanen R (1990). The role of attention in auditory information processing as revealed by event-related potentials and other brain measures of cognitive function. Behavioural Brain Sciences.

[CIT0024] Norman S. B, Stein M. B, Davidson J. R. T (2007). Profiling posttraumatic functional impairment. Journal of Nervous and Mental Disease.

[CIT0025] Olofsson J.K, Nordin S, Sequiera H, Polich J (2008). Affective picture processing: An integrative review of ERP studies. Biological Psychology.

[CIT0026] Pizzigalli D, Regard M, Lehmann D (1999). Rapid emotional face processing in the human right and left brain hemispheres: An ERP study. Neuroreport.

[CIT0027] Polich J, Kok A (1995). Cognitive and biological determinants of P300: An integrated review. Biological Psychology.

[CIT0028] Stemmler G, Ave T, Wacker J (2007). Anger and fear: Separable effects of emotion and motivational direction on somatovisceral responses. International Journal of Psychophysiology.

[CIT0029] Utama N. P, Takemoto A, Koike Y, Nakamura K (2009). Phased processing of facial emotion: An ERP study. Neuroscience Research.

[CIT0030] Williams L. M, Liddell B. J, Rathjen J, Brown K. J, Gray J, Phillips M (2004). Mapping the timecourse of nonconscious and conscious perception of fear: An integration of central and peripheral measures. Human Brain Mapping.

